# Vogt-Koyanagi-Harada Syndrome: A Diagnostic Conundrum

**DOI:** 10.7759/cureus.20138

**Published:** 2021-12-03

**Authors:** Anila Hussain, Ritu Khurana

**Affiliations:** 1 Internal Medicine, Crozer-Chester Medical Center, Upland, USA; 2 Rheumatology, Crozer-Chester Medical Center, Upland, USA

**Keywords:** vkh disease, vogt-koyanagi-harada disease, uveitis, auto immune, acute blindness

## Abstract

Vogt-Koyanagi-Harada disease is a vision-threatening autoimmune disease mediated by adaptive immune responses via T helper (Th) 1 and Th17 cell activation. The disease often starts with a flu-like illness followed by eye pain, headache, and dizziness later evolving into vision loss bilaterally. Other symptoms may include vitiligo and hearing loss. Diagnostic criteria include exclusion of other eye diseases, no history of recent penetrating eye trauma or surgery, bilateral ocular involvement with evidence of diffuse choroiditis, auditory and neurological findings (tinnitus and meningismus), and skin findings including depigmentation or alopecia. Retinal examination reveals bilateral uveitis with choroidal thickening (which may be seen as a sub-retinal fluid collection or serous retinal detachment). Treatment includes corticosteroid therapy with the addition of biological and immunosuppressive medications as needed to suppress the disease activity and ensure symptomatic improvement.

## Introduction

Vogt-Koyanagi-Harada syndrome (VKH syndrome) is a rare disease of autoimmune origin that affects multiple organ systems of the body including eyes, ears, skin, and meninges. Diagnostic criteria include exclusion of other eye diseases, no history of recent penetrating eye trauma or surgery, bilateral ocular involvement with evidence of diffuse choroiditis, auditory and neurological findings (tinnitus and meningismus), and skin findings including depigmentation or alopecia [1]. The most specific feature of the disease includes bilateral granulomatous pan-uveitis resulting in rapid loss of vision leading to blindness. This disease is often preceded by a flu-like illness followed by common presenting symptoms of eye pain, headache, and dizziness. Eye pain is often followed by rapid vision loss in one eye followed by the other, or both eyes at the same time. Other symptoms include hearing loss, skin depigmentation, and hair loss [2]. Disease pathogenesis is known to be autoimmune in nature as per the latest studies. Evidence has also shown the contribution of genetic factors including VKH specific risk factors (human leukocyte antigen [HLA]-DR4), and general risk factors for immune-mediated diseases (interleukin-23 receptor [IL-23R]), dysfunction of immune responses, including the innate and adaptive immune system included Th1 and Th17 cell activation against own antigens [3]. Environmental triggering factors are also found to be involved in the development of VKH disease.

## Case presentation

A 30-year-old Hispanic female with a past medical history of depression and anxiety presented with headache, blurring of vision, and photophobia for two weeks prior to presentation. Symptoms were described as severe, and progressively worsening. The vision was described as a distortion of objects on near-vision or “squiggly lines”. The patient was initially seen by a neurologist soon after presentation and symptoms were attributed to migraines and the patient was recommended to see neurology outpatient or ophthalmology if symptoms continue to worsen. The patient continued to have worsening photophobia along with blurring of vision followed by a significant decrease in visual activity, hence, inpatient ophthalmology was consulted. Eye exam showed cream-colored placoid findings with initial concern for acute posterior multifocal placoid pigment epitheliopathy. The patient was initially ruled out for a new-onset stroke with a CT scan of the head and perfusion scans which were negative. Later, the patient had persistent symptoms and progressive visual loss leading to a reassessment of stroke via brain MRI, which did not show any evidence of intracranial hemorrhage, ischemia, or demyelinating disease. The patient had symptoms of photophobia and neck rigidity prompting a lumbar puncture to rule out meningitis or intracranial hypertension. Lumbar puncture results showed normal cerebrospinal fluid (CSF) opening pressure and normal CSF analysis. Infectious workup for uveitis including syphilis, Lyme, and tuberculosis was found negative. The patient was seen by an ophthalmologist and a detailed eye examination and Fluorescein Angiography (FA) was performed that showed multiple areas of depigmentation with serous detachment associated with choroidal thickness. Sub-retinal fluid with choroidal thickening close to the macula on the right eye was also detected. Visual acuity was 20/50 bilaterally which worsened on the subsequent examinations. In the absence of alternative ocular pathology or penetrating trauma to the eye, the presence of bilateral ocular involvement with serous detachment and sub-retinal fluid presence along with mild alopecia and neurological symptoms led to the diagnosis of Vogt-Koyanagi-Harada Syndrome. After confirmation of diagnosis, the patient was started on prednisone initially 50mg daily, which did not result in significant improvement in a few weeks, so the dosage was increased to 100mg daily. The patient’s symptoms stabilized temporarily, but upon no further improvement, it was decided to administer adalimumab and methotrexate. Adalimumab was later switched to infliximab after three months of therapy secondary to transaminitis and persistent symptoms. The patient reported improvement in symptoms on the new regimen and prednisone tapering was started. Currently, the patient is maintained on 20mg prednisone, 12.5mg methotrexate and, infliximab 8mg/kg. The patient was discharged after a combined treatment suggested by rheumatology and ophthalmology care providers and advised to appear for a three-monthly follow-up at both specialties.

## Discussion

Ancillary tests that can be used in the diagnosis of VKH syndrome include FA, B-scan ultrasonography, and lumbar puncture [4,5]. Diagnosis of VKH syndrome should prompt rapid treatment with corticosteroids. However, secondary to the high side effect profile of steroids, the introduction of immunosuppressants is often needed [6,7]. Some of the immunosuppressant medications that can be used include methotrexate and cyclosporine [8]. For refractory cases, biological agents are often a good addition. Some commonly used agents include infliximab, adalimumab, and rituximab [9-13]. Our patient was initially started on prednisone with the later addition of adalimumab and methotrexate. Adalimumab was eventually switched to infliximab and the patient reported improvement in symptoms with a combination of prednisone, methotrexate, and infliximab.

## Conclusions

Vogt-Koyanagi-Harada syndrome is a rare and serious vision-threatening disease that is often seen in Asians, Native Americans, Middle Eastern, and Hispanic populations. In absence of apparent ocular abnormalities/trauma explaining symptoms and negative stroke and meningitis work up, prompt diagnosis by eye exam should be done to ensure early diagnosis and treatment to prevent permanent visual loss. Steroids are the mainstay of treatment for the disease with the addition of biological agents often necessary to prevent worsening of the disease and to avoid permanent damage. Steroids can be tapered after the addition of biological agents to avoid long-term complications of the steroids.

## References

[1] Read RW, Holland GN, Rao NA (2001). Revised diagnostic criteria for Vogt-Koyanagi-Harada disease: report of an international committee on nomenclature. Am J Ophthalmol.

[2] Fang W, Yang P (2008). Vogt-Koyanagi-Harada syndrome. Curr Eye Res.

[3] Bušányová B, Tomčíková D, Gerinec A (2013). Vogt-Koyanagi-Harada syndrome in children - a case report (Article in Czech). Cesk Slov Oftalmol.

[4] da Silva FT, Damico FM, Marin ML (2009). Revised diagnostic criteria for Vogt-Koyanagi-Harada disease: considerations on the different disease categories. Am J Ophthalmol.

[5] Khairallah M, Zaouali S, Messaoud R, Chaabane S, Attia S, Ben Yahia S, Hmidi K (2007). The spectrum of Vogt-Koyanagi-Harada disease in Tunisia, North Africa. Int Ophthalmol.

[6] Urzua CA, Velasquez V, Sabat P (2015). Earlier immunomodulatory treatment is associated with better visual outcomes in a subset of patients with Vogt-Koyanagi-Harada disease. Acta Ophthalmol.

[7] Lai TY, Chan RP, Chan CK, Lam DS (2009). Effects of the duration of initial oral corticosteroid treatment on the recurrence of inflammation in Vogt-Koyanagi-Harada disease. Eye (Lond).

[8] Andreoli CM, Foster CS (2006). Vogt-Koyanagi-Harada disease. Int Ophthalmol Clin.

[9] Cordero-Coma M, Yilmaz T, Onal S (2013). Systematic review of anti-tumor necrosis factor-alpha therapy for treatment of immune-mediated uveitis. Ocul Immunol Inflamm.

[10] Chen L, Yang P, Zhou H (2008). Diminished frequency and function of CD4+CD25 high regulatory T cells associated with active uveitis in Vogt-Koyanagi-Harada syndrome. Invest Ophthalmol Vis Sci.

[11] Khalifa YM, Bailony MR, Acharya NR (2010). Treatment of pediatric Vogt-Koyanagi-Harada syndrome with infliximab. Ocul Immunol Inflamm.

[12] Niccoli L, Nannini C, Cassarà E, Gini G, Lenzetti I, Cantini F (2009). Efficacy of infliximab therapy in two patients with refractory Vogt-Koyanagi-Harada disease. Br J Ophthalmol.

[13] Zmuda M, Tiev KP, Knoeri J, Héron E (2013). Successful use of infliximab therapy in sight-threatening corticosteroid-resistant Vogt-Koyanagi-Harada disease. Ocul Immunol Inflamm.

